# Demographic and clinical features of neuromyelitis optica: A review

**DOI:** 10.1177/1352458515572406

**Published:** 2015-06

**Authors:** L Pandit, N Asgari, M Apiwattanakul, J Palace, F Paul, MI Leite, I Kleiter, T Chitnis, the GJCF International Clinical Consortium & Biorepository for Neuromyelitis Optica

**Affiliations:** KS Hegde Medical Academy, Nitte University, Mangalore, Karnataka, India; Neurobiology, Institute of Molecular Medicine, University of Southern Denmark, and Department of Neurology, Vejle Hospital, Denmark; Prasat Neurological Institute Bangkok, Thailand; Department of Clinical Neurology, Oxford University Hospitals, Oxford, UK; Neuro Cure Clinical Research Center and Clinical and Experimental Multiple Sclerosis Research Center, Department of Neurology, Charité University Medicine Berlin, Berlin, Germany; Department of Clinical Neurology, Oxford University Hospitals, Oxford, UK; Department of Neurology, St. Josef-Hospital, Ruhr-University Bochum, Bochum, Germany; Department of Neurology, Brigham and Women’s Hospital and Massachusetts General Hospital, Boston, USA

**Keywords:** NMO, epidemiology, demographics, incidence, prevalence

## Abstract

The comparative clinical and demographic features of neuromyelitis optica (NMO) are not well known. In this review we analyzed peer-reviewed publications for incidence and prevalence, clinical phenotypes, and demographic features of NMO. Population-based studies from Europe, South East and Southern Asia, the Caribbean, and Cuba suggest that the incidence and prevalence of NMO ranges from 0.05–0.4 and 0.52–4.4 per 100,000, respectively. Mean age at onset (32.6–45.7) and median time to first relapse (8–12 months) was similar. Most studies reported an excess of disease in women and a relapsing course, particularly in anti-aquaporin 4 antibody (anti AQP4-IgG)-positive patients. Ethnicity may have a bearing on disease phenotype and clinical outcome. Despite limitations inherent to the review process, themes noted in clinical and demographic features of NMO among different populations promote a more global understanding of NMO and strategies to address it.

## Introduction

Neuromyelitis optica (NMO) is an autoimmune inflammatory disorder of the central nervous system that predominantly targets the optic nerve and spinal cord. Until recently, NMO was thought to be a clinical variant of multiple sclerosis (MS). However, clinical features, neuroimaging, immunological, and histopathological characteristics have now been identified which distinguish NMO from MS. Clifford and Gault summarized the salient features of the disease in 1894, though descriptions of the disease appeared much earlier. The typically long spinal cord segmental involvement, longitudinally extensive transverse myelitis (LETM) and neutrophilic pleocytosis in the cerebrospinal fluid (CSF) became incorporated into the first Wingerchuk diagnostic criteria for NMO in 1999.^1^ Subsequently, the discovery of anti AQP4-IgG^2^ led to the revised Wingerchuk criteria in 2006.^3^ The term “NMO spectrum disorders”^4^ (NMOSD) encompasses forms of NMO that do not satisfy the 2006 criteria. Examples include isolated unilateral or simultaneous bilateral or recurrent optic neuritis (ON); isolated or recurrent transverse myelitis (TM); typical NMO brain lesions (hypothalamus, corpus callosum, brainstem, periventricular) with or without detectable anti AQP4-IgG autoantibody. Thus, the diagnostic criteria have been refined and the spectrum of clinical manifestations in NMO has expanded in recent years.

NMO occurs worldwide in diverse races and cultures; however, the global incidence and prevalence of this disease is incompletely characterized. More specifically, it is unclear whether disease frequency, clinical course, severity, and gender predominance vary between geographical regions. Obtaining epidemiological and demographic data for a relatively uncommon disorder like NMO is challenging. Very few published reports of NMO have appeared from emerging nations. Access to magnetic resonance imaging (MRI) centers varies, as do laboratory facilities for CSF analysis and anti AQP4-IgG detection. Hence, timing of the MRI after an attack of myelitis and blood sampling for anti AQP4-IgG testing may vary. Furthermore, lack of uniformity of survey methods, disease definitions, and diagnostic criteria complicate interpretation of the literature.

A small proportion of patients with NMO are seronegative for anti AQP4-IgG, and may represent a heterogeneous spectrum of NMO or disease mimics (including MS and other diseases such as sarcoidosis and paraneoplastic disorders). Thus, studies that include all clinically defined NMO patients are difficult to compare with those selecting only anti AQP4-IgG-positive patients. In addition, assays for detection of anti AQP4-IgG vary with regard to their sensitivity and specificity. Although most contemporary assays perform well in a high-probability patient, when used for population screening the seropositive rate can vary by two-fold or greater between assays of different specificity.^5,6^ The initial AQP4-IgG immunofluorescence assay that was developed is the least sensitive.^2,6^ The cell-based assay (CBA), although labor intensive and less suited to mass screening, is most sensitive and specific for use in clinical diagnosis.^6,7^ Thus, differences in diagnostic criteria and anti AQP4-IgG assays may account for heterogeneity in epidemiologic data, and may explain some of the observed discrepancies in interpretation.

The goal of this review article is to assess major epidemiological and hospital-based studies to estimate incidence and prevalence of NMO in different geographic regions, and to provide insights into the differences and similarities of demographic and clinical features of NMO among diverse populations.

## Methods

In this paper, we reviewed the published reports of population-based studies, hospital-based retrospective chart reviews, and data from NMO registries. We queried the PubMed database from the years 1999–2014. The search terms used included “neuromyelitis optica”, “Devic’s disease”, “incidence”, “prevalence”, “epidemiology”, “demography” and “clinical features”. In total, 48 studies were examined. Of these, seven population-based epidemiological studies (Table 1) and nine hospital-based studies were included for comparative analysis of clinical and demographic features (Table 2). All studies that were included had utilized Wingerchuck 1999 or 2006 criteria, and results of anti AQP4 - IgG testing were available. Lastly, to integrate a global demographic perspective, selected data obtained from the Atlas of MS 2013 (www.atlasofms.org) were also included in the analysis.

**Table 1.** Comparative Incidence and prevalence of NMO from recently published studies.

Country	Diagnostic criteria	Source of data	No of cases/population at risk	Ethnicity	Crude incidence/100,000	Crude prevalence/100,000	F/M
French West Indies Cabre et al.,^9^ 2007/2009	Wingerchuk 1999, 2006	Hospitals, clinics, neurologists, other specialists, registry, patient association	20/ 683,000	Afro-Caribbean Hispanic	0.19 (0.15–0.23)	2.53	All female
Cuba Cabrera Gomez et al.,^10^ 2009	Wingerchuk 1999	Hospitals, clinics, other specialists, neurologists, clinical trial data, MS data base, media	11/11,177,743	Caucasian Afro-Caribbean	0.053 (0.04–0.07)	0.54 (0.39–0.69)	7.3:1
Denmark Asgari et al.,^8^ 2011	Wingerchuk 2006	National Registry, Hospitals, MS-clinics, neurologists, other specialists, administrative databases	42/952,000 NMO=36, NMOSD=6	99% Caucasian	0.04	4 (3.1–5.7)	2.8:1
Wales Cossburn et al.,^13^ 2012	Wingerchuk 2006	Hospital records, Neurologists, Regional database, Lab records	14/717,572 NMO=11, NMOSD=3	100% Caucasian	–	1.96 (1.22–2.97)	6:1
Japan Houzen et al.,^14^ 2012	Wingerchuk 2006	Inpatient and outpatient records of MS-related institutions	3/352,353	Japanese	NA	0.9 (0.2–2.5)	All female
UK Jacob et al.,^11^ 2013	Wingerchuk 2006	Hospital records, regional general hospital records, lab records	8/1,145,322 NMO=5 NMOSD=3	Caucasian 7 (88%) African 1(12%)	0.08 (0.03–0.16)	0.72 (0.3–0.16)	3.5:1
India Pandit et al.,^15^ 2014	Wingerchuk 2006	Disease registry, inpatient and outpatient records of teaching hospitals and clinics, MRI centers	11/ 419,306	South Indians	NA	2.6	1.2:1

**Table 2.** Clinical and demographic features of NMO^#^.

Country/ study	Wingerchuk diagnostic criteria	Cases selected	Total cases & gender (F/M)	Ethnicity	Mean Age at onset & range	Median time to 1^st^ relapse (months)	First attack	AQP4* IgG +ve	Median follow-up	Median EDSS	Mortality disease related
French West Indies Cabre et al.,^9^ 2007/2009	Wingerchuk 1999, 2006	NMO	96 (88F/8M)	Hispanic Afro-Caribbean	29.5 (11–74)	11.5 (1–300)	NA	32%	9.5 (1–40) years	NA	24 (25%)
Brazil Bichuetti,^20^ 2009	1999, 2006	NMO relapsing	41 (29F/ 12M)	NA	32.6 (20–60)	NA	TM 41% ON34%ON+TM 24%	41%	52 (7–200) months	NA	4 (14.3%)
France Collongues et a1.,^16^ 2010	2006	NMO, NMOSD	125 (94F/31M)	White=87% other=13%	34.7 (4–66)	12 (30.8–43.1)	TM 45.6% ON 36.8%	54%	8.7 (0.1–39.5) years	NA	4 (3.2%)
Denmark Asgari et al.,^8^ 2011	2006	NMO NMOSD	42 (31F/11M)	White=99%	35.6 (15–64)	NA	TM 59.5% ON 35.7%	62.0%	6.5 (2–10) years	6.5 (1.0–9)	3 (7.1%)
Germany Jarius et al.,^17^ 2012	2006	NMO, NMOSD	175 (150F/25M)	White =100%	39 (10-81)	8.5 (1–216)	ON 58% ON+TM 8 %	78.3%	4.8 (0–390) months	6.5 (1.5–10)	5 (2.9%)
USA Mealy et al.,^19^ 2012	2006	NMO, NMOSD	187(162F/25M)	White=89 AA=69 other=29	40	NA	TM 50.2% ON 35.3%	66.6%	5.5 (0.2–38) months	NA	NA
Korea Kim et al.,^22^ 2012	2006	NMO, NMOSD	106 (97F/9M)	Korean=100%	NA	5 (1–74)	NA	100%	7.0 (1–24) years	NA	NA
India Pandit et al.,^21^ 2013	2006	NMO, NMOSD	70(30F/40M	South Indians-100%	37.5 (12–65)	12 (4–96)	NA	39%	4.5 (1–13) years	5.5 (1.5–10)	12 (17%)
Austria Aboul-Enein et al.,^12^ 2013	1999, 2006 (all seropositive )	NMO, NMOSD	73 (63/10)	White=100%	45.7 (12.3–79.6)	NA	ON 54.2% TM 41.7% ON+TM 4.2%	97% (71/73)	NA	NA	NA
Spain Höftberger et al.,^18^ 2014	2006	NMO	48 (41F/7M)	White=94% Other=6%	38 (18–73.5)	8.3 (1–144)	TM 36%; ON 29%; ON+TM 33%	81%	69 (1–415) months	3.8 (0–10)	2 (4.2%)

AF: African; AS: Asian; AA: African American; ON: optic neuritis; TM: transverse myelitis; ARR: annualized relapse rate; NA: not available.

* percentage of patients who were AQP4-IgG positive among those tested.

# Used either Wingerchuk 1999 or 2006 criteria, all others used Wingerchuk 2006 alone.

## Population-based studies

While data from population-based studies are likely to be more robust, several factors make direct comparisons across such studies challenging. For example, variability in the source data (e.g. clinic, health insurance data, or population based), diagnostic criteria, inclusion of seronegative individuals, definition of NMOSD, and the particular anti AQP4-IgG assay used are important factors undoubtedly influencing the results. The ethnic mix of the populations studied may also influence the prevalence rates. In addition, most published studies have focused on adults.

## Incidence

There have been several recent studies from Denmark, West Indies, Cuba, the UK, and Austria from which unadjusted incidence rates (per 100,000) could be calculated (Table 1).^8–12^ In a Danish population comprising 99% whites, an incidence rate of 0.40 (95%CI 0.30–0.54) was found to be the highest among the five studies included.^8^ A French West Indies study^9^ found that the age and sex-standardized incidence was 0.22 (95%CI 0.04–0.38), and remained consistent between 1992 and 2007. While the latter study included only NMO cases in which Wingerchuk criteria were fully met, the Danish study included all individuals carrying the diagnosis of NMO, regardless of whether they met these criteria. Thus, selection criteria may influence incidence rates of the disease. A Cuban study included white subjects of Spanish descent, African, and those of mixed racial origins.^10^ Here, an overall incidence rate of 0.05 was observed, and was similar across ethnic groups, but varied from 0.12–1.76 across geographical regions of Cuba. The incidence rate in Merseyside, UK, a population consisting of 88% whites, was found to be 0.08 (95%CI 0.03–0.16).^11^ An Austrian study^12^ reported an incidence rate of 0.05 (95% CI 0.01–0.31), in an all-white population.

## Prevalence

Several studies have measured prevalence (per 100,000) in NMO. The prevalence was 4.4 in Denmark^8^ (95%CI 3.1–5.7), 1.96 among the white population of Wales,^13^ 0.72 in Merseyside, UK,^11^ 2.3 in the French West Indies^9^ and 0.52^10^ in Cuba. The vast majority of patients in an NMOSD cohort in Austria^12^ (71/73, all whites) were seropositive. The prevalence rate of NMO was 0.77 per 100,000.^12^ Prevalence data are also available from Japan and India. In Japan, a prevalence rate of 0.9 per 100,000 (95%CI 0.2–2.5) was determined,^14^ while in southern India it was 2.7/100,000.^15^ Collectively, these studies suggest a relatively wide range in prevalence, including differences in geographic and ethnic cohorts. The Danish study was the only one that included limited forms of NMO.

## Hospital-based studies

Nine hospital-based observational studies were identified (Table 2), four from Europe,^12,16–18^ one each from the USA,^19^ Brazil,^20^ Austria,^12^ India,^21^ and Korea.^22^ All of these studies used Wingerchuk 2006 diagnostic criteria except the Spanish^18^ study (which used 1999 and 2006 criteria). Median time to second attack was 8–12 months (range 1–216 months). Mortality ranged from 2.9–25% and was disease related in the majority of cases. Analysis of registry-based data suggests that the proportion of patients with NMO among all demyelinating disorders varies significantly among different geographic regions: 1.2% in Italy,^23^ 1–2% in the USA,^19^ 13.7% in India,^21^ and 39.3% in Thailand.^24^ The study from Thailand underscores the need for supportive radiological criteria that justify the clinical diagnosis. Several anti AQP4-IgG seropositive patients with a diagnosis of MS, in retrospect had radiological and clinical features of NMO.

## Effect of age

There are geographic differences with respect to NMO and patient age. From prevalence studies, median age at onset was found to be 30.5 years in Cuba,^10^ 39.5 years in South East Wales,^13^ 30 years in Denmark,^8^ and 55.2 years in Austria.^12^ These differences may reflect variations in case ascertainment and diagnostic criteria, access to medical care, and/or ethnicity. In the Cuban study, blacks were older than non-blacks at disease onset; however, there were no differences between other subgroups.^10^ The mean age of onset of NMO ranged from 32.6–45.7 years in major case series (Table 2). In a study comparing seropositive individuals (based on the CBA) in the UK, and Japan, Afro-Caribbeans experienced earlier disease onset than white and Asian patients.^24^ Pediatric onset is rare, with <5% of cases presenting prior to age 18 in two studies.^25,26^ In contrast, late-onset NMO/NMOSD, defined as onset age >50 years, accounted for 108/430 (25%) in a large multicenter cohort study.^27^ Compared with patients with adult-onset NMO, those with pediatric NMO more frequently have brain involvement, experience milder locomotor disability, and develop significantly earlier visual disability.^25^ In a German study that contrasted clinical presentation of seropositive and negative NMO patients, the proportion of anti-AQP4-IgG-positive patients appeared to increase with age; however, mean age at onset did not differ among seronegative and seropositive patients.^17^ The largest study assessing serological status and age is that from the Mayo clinic, which compared the demographics of over 50,000 patients referred for NMO-IgG testing.^26^ Similar to the German study,^17^ the proportion of seropositive females was shown to increase with age, and was highest in the >65 year-old group, accounting for 12% of the entire seropositive cohort.

## Gender ratio

As with many autoimmune diseases, NMO and NMOSD are more common in females, ranging from 66–88% of the patient population.^8,9,16^ One study reported an 89% female predominance when including only cases with relapsing NMO. Even prior to the discovery of anti AQP4-IgG, relapsing NMO was known to be more prevalent in females (83%), whereas a more equal female/male distribution was detected in monophasic disease (48%). Anti AQP4-IgG positivity was associated with relapsing disease with rates of 81–91%,^17,28^ particularly when CBA is used. Among patients with LETM, females represent 86% of anti AQP4-IgG seropositive compared with 44% of seronegative individuals.^29^ The variation across studies may be influenced by limited patient numbers in those conducted within specialized centers. Moreover, there may be racial and geographical effects. For example, in a comparative study from different populations^24^ females represented 82% of patients in whites from the UK, compared with 75% in Afro-Caribbeans and 98% in a Japanese cohort. In an all-white Austrian cohort, the female ratio was 87% for all AQP4-IgG-positive patients^12^ while it reached 100% in a French West Indian cohort.

## Disease onset, course and severity

Several multicenter registry-based observational, retrospective studies (Table 2) provide information about clinical course and disease severity. Some common findings emerged from these studies. The presenting symptom was usually either ON or TM, especially among seropositive patients. Simultaneous TM and ON, bilateral ON, and monophasic course were relatively more common presentations among seronegative patients.^16,17,29^ Males and females were similarly affected in this subgroup.^17,29^ Age at disease onset appears to have a bearing on disability type. In a comparative study, Kitley et al.^25^ found that patients in the UK had young-onset disease commonly presenting with ON and had a high risk of visual disability. An older age of onset was significantly associated with motor disability, in both Japanese and UK cohorts. Jarius et al.^17^ have further identified distinct clinical features for NMO patients based on anti AQP4-IgG serological status. Seropositive women had more severe clinical attacks than males in this cohort, as evidenced by high lesion load in the spinal cord and other types of co-existing autoimmunity. Overall, seropositive and seronegative patients did not differ in terms of age of onset, time to relapse, relapse rates, rate of Expanded Disability Status Scale (EDSS) progression, and mortality rate. Irrespective of anti AQP4-IgG serological status, predictors of outcome included development of tetraparesis during the first attack of TM, and multiple attacks of TM in the first year. Distribution of brain lesions was different depending on anti AQP4-IgG status. Periaqueductal grey matter, hypothalamic, and area postrema lesions may be more often seen in seropositive patients.^30^ In the Danish study, NMO-IgG seropositive patients frequently developed brainstem lesions and LETM during the decade of follow-up.^31^ A study from Barcelona, which focused on LETM, found that EDSS at presentation was predictive of the recurrence risk and disability.^32^

## Ethnicity and disease course

Ethnicity appears to be an influential factor in NMO and NMOSD. In the Cuban study^10^ patients of African origin were older, had more relapses, abnormalities in brainstem-evoked potentials, and acquired more lesions on brain MRI than comparator patients. African Americans in the US^18^ had significantly more brainstem MRI abnormalities than white Americans. Kitley and colleagues^25^ compared the disease course and prognostic factors for seropositive NMO patients in the UK (*n*=59) and Japan (*n*=47). In the UK group, 45/59 were white, 12/59 were Afro-Caribbean, and two were Asian. Age of onset and ethnicity were associated with outcome. Young white patients presented with ON, and had greater visual disability. In contrast, older patients of both ethnicities presented with myelitis and greater motor disability. The Afro-Caribbean subgroup was significantly younger at onset than both white and Japanese patients (mean 28.0 ± 13.1 years and 44.9 ± 17.2 years, respectively). Initial events more commonly included those of brain/brainstem in Afro-Caribbean patients compared with white and Japanese, and uniformly had relapsing NMO compared with 80% in the other ethnicities. Annualized relapse rate among Afro-Caribbean patients was comparable with that of white patients but higher than that of the Japanese. Visual disability was more severe in non-white patients. In contrast, white patients had a later age of onset and worse motor outcomes. In the study conducted in Cuba and French West Indies,^9,10^ lack of recovery after the first attack and a higher rate of attacks during the first year of disease were strong independent prognostic factors of mortality.

## Population genetics

Initial epidemiological studies have suggested ethnicity-based differences in genetics of NMO patients. However, no specific genetic determinants of NMO disease have thus far been identified. The genetic basis for these ethnic differences is not well understood, but the human leukocyte antigen (HLA) system remains an obvious candidate. Studies from Japan have analyzed the clinical and genetic features of the optico-spinal form of multiple sclerosis (OSMS), a disease entity which may overlap with NMO. In that population, conventional MS was associated with HLA-DRB1*1501 whereas OSMS/NMO was associated with HLA DPB1*0501.^33^ Other population studies of HLA in NMO indicate that the DRB1*0301^34–36^ and DRB1*10^37^ alleles are associated with increased risk.

Familial NMO has been described. In the largest study, Matiello et al.^38^ identified 12 families (five Asian, four Latino, two African, one white) with a total number of 25 affected individuals, 19 of whom were AQP4-IgG positive. The role of environmental influences in the etiopathogenesis of NMO has not been established. Migration studies are needed to explore whether risk is mutable with migration and how this relates to age at migration.

## Insights into NMO epidemiology from the Atlas of MS Database

In 2013, a questionnaire designed to update the MS global dataset added a number of queries relating to NMO, including prevalence, incidence, gender ratio, mean age of onset, diagnostic criteria used, and therapeutic options. This questionnaire received notifications of 10,567 cases obtained from 40 of 107 countries participating in the survey. Roughly one-third of the patients reported were from Japan (3500), creating potential bias. Data on prevalence (expressed as cases per 100,000) were available from 35 countries. In temperate zones, Central European countries had a prevalence ranging from 0.23 in Belgium to 2.0 in Norway, while Denmark (4.4) and The Netherlands (5) had comparatively high prevalence. By comparison, NMO prevalence among the Japanese population was 2.8. South Africa and Paraguay had the highest prevalence (5), followed by French West Indies (4.2). In the Middle East, the projected figures ranged from 0.16–2 and in South East Asia from 0.43–2.7. These widely disparate reports, particularly among European countries, may have resulted from differing methodologies involved in the collection of source data. No data were available from most regions of the African continent, Eastern Europe, parts of Central America, and South Asia. In each population studied patients were predominantly female, with ratios ranging from 1.0 in Ireland to 10.1 in Paraguay. Overall the mean age of onset ranged from 25.0–45.7 years. The MS database provided information from a variety of countries across the globe. In most instances, the findings were not supported by published literature and were often sourced from single or few individuals with an interest in the subject. Resource-poor countries had few neurologists and limited MRI centers, and diagnostic NMO-IgG testing was limited or non-existent. These factors hampered the reliability of the survey.

## Conclusions

NMO epidemiology has largely been based on relatively small populations and data from tertiary hospitals. Many of these initial studies have yielded inconsistent findings. Confounding factors include the use of non-standardized diagnostic criteria, variability in assays for anti-AQP4 antibody, inconsistent inclusion of NMO-IgG seronegative patients, small study cohorts, and potential for selection bias. These factors limit the comparative assessment and statistical power of epidemiologic studies in NMO and NMOSD. While differences in ethnicity appear to impact clinical disease course and NMO-IgG status, recent studies have disclosed similarities in incidence and prevalence of NMO. Female predominance is commonly seen and appears congruent with NMO-IgG seropositive status, which itself associates with a relapsing phenotype. HLA haplotype associations have been hypothesized, but the genetic basis for differences in risk, severity, therapeutic efficacy, or clinical outcomes remains uncertain. Larger adequately powered epidemiological studies applying standardized definitions and methodologies are needed before specific conclusions can be drawn. The advent of more specific diagnostic criteria, improved diagnostic assays and methods, and a better understanding of the basic immunology of NMO and NMOSD are likely to advance these goals. In turn, these steps should improve consistency in diagnosis and enhance study cohorts for the most informative clinical trials for NMO.

## References

[1] Wingerchuk DM Hogancamp WF O’Brien PC The clinical course of neuromyelitis optica ( Devic’s syndrome). Neurology 1999; 53: 1107–1114. 10496275 10.1212/wnl.53.5.1107

[2] Lennon V Wingerchuk D Kryzer T A serum autoantibody marker of neuromyelitis optica: Distinction from multiple sclerosis. Lancet 2004; 36445: 2106–2112. 15589308 10.1016/S0140-6736(04)17551-X

[3] Wingerchuk DM Lennon VA Pittock SJ Revised diagnostic criteria for neuromyelitis optica. Neurology 2006; 66: 1485–1489. 16717206 10.1212/01.wnl.0000216139.44259.74

[4] Wingerchuk DM Lennon VA Lucchinetti CF The spectrum of neuromyelitis optica. Lancet Neurol 2007; 6: 805–815. 17706564 10.1016/S1474-4422(07)70216-8

[5] Pittock SJ Lennon VA Bakshi N Seroprevalence of aquaporin-4-IgG in a Northern California population representative cohort of multiple sclerosis. JAMA Neurol. 2014; 71: 1433–1436. 25178362 10.1001/jamaneurol.2014.1581

[6] Waters PJ McKeon A Leite MI Serologic diagnosis of NMO: A multicenter comparison of aquaporin-4-IgG assays. Neurology 2012; 78: 665–671. 22302543 10.1212/WNL.0b013e318248dec1 PMC3286228

[7] Jarius S Wildemann B Aquaporin-4 antibodies (NMO-IgG) as a serological marker of neuromyelitis optica: A critical review of the literature. Brain Pathol 2013; 23: 661–683. 24118483 10.1111/bpa.12084 PMC8028894

[8] Asgari N Lillevang ST Skejoe HP A population-based study of neuromyelitis optica in Caucasians. Neurology 2011; 76: 1589–1595. 21536639 10.1212/WNL.0b013e3182190f74 PMC3269768

[9] Cabre P Environmental changes and epidemiology of multiple sclerosis in the French West Indies. J Neurol Sci 2009; 286: 58–61. 19520388 10.1016/j.jns.2009.04.039

[10] Cabrera-Gomez JA Kurtzke JF Gonzalez-Quevedo A An epidemiological study of neuromyelitis optica in Cuba. J Neurol 2009; 256: 35–44. 19224310 10.1007/s00415-009-0009-0

[11] Jacob A Panicker J Lythgoe D The epidemiology of neuromyelitis optica amongst adults in the Merseyside county of United Kingdom. J Neurol 2013; 260: 2134–2137. 23689970 10.1007/s00415-013-6926-y

[12] Aboul-Enein F Seifert-Held T Mader S Neuromyelitis optica in Austria in 2011: To bridge the gap between neuroepidemiological research and practice in a study population of 8.4 million people. PLoS One 2013; 8: e79649. 24223985 10.1371/journal.pone.0079649 PMC3818238

[13] Cossburn M Tackley G Baker K The prevalence of neuromyelitis optica in South East Wales. Eur J Neurol 2011; 19: 655–659. 21967235 10.1111/j.1468-1331.2011.03529.x

[14] Houzen H Niino M Hirotani M Increased prevalence, incidence, and female predominance of multiple sclerosis in northern Japan. J Neurol Sci 2012; 323: 117–122. 22995683 10.1016/j.jns.2012.08.032

[15] Pandit L Kundapur R Prevalence and patterns of demyelinating central nervous system disorders in urban Mangalore, South India. Mult Scler J 2014; 20: 1651–1653. 10.1177/1352458514521503 24493471

[16] Collongues N Marignier R Zéphir H Neuromyelitis optica in France: A multicenter study of 125 patients. Neurology 2010; 74: 736–742. 20194912 10.1212/WNL.0b013e3181d31e35

[17] Jarius S Ruprecht K Wildemann B, for the German Neuromyelitis Optica Study Group (NEMOS). Contrasting disease patterns in seropositive and seronegativeneuromyelitis optica: A multicentre study of 175 patients. J Neuroinflammation 2012; 9: 14. 22260418 10.1186/1742-2094-9-14 PMC3283476

[18] Höftberger R Sepúlveda M Armangue T Antibodies to MOG and AQP4 in adults with neuromyelitis optica and suspected limited forms of the disease. Mult Scler 2014; 10.1177/1352458514555785 [Epub ahead of print]. PMC4824843 25344373

[19] Mealy M Wingerchuck D Greenberg B Epidemiology of neuromyelitis optica in the United States, a multicenter analysis. Arch Neurol 2012; 69: 1176–1180. 22733096 10.1001/archneurol.2012.314

[20] DB Bichuetti DB Oliveira EML Souza NA Neuromyelitis optica in Brazil: A study on clinical and prognostic factors. Mult Scler 2009; 15: 613–619. 19299436 10.1177/1352458508101935

[21] Pandit L Mustafa S Optimizing the management of neuromyelitis optica and spectrum disorders in resource poor settings: Experience from the Mangalore demyelinating disease registry. Ann Ind Academy Neurol 2013; 16: 572–576. 10.4103/0972-2327.120474 PMC3841603 24339582

[22] Kim SH Kim W Li XF Clinical spectrum of CNS aquaporin-4 autoimmunity. Neurology 2012; 78: 1179–1185. 22459676 10.1212/WNL.0b013e31824f8069

[23] Bizzoco E Lolli F Repice AM Prevalence of neuromyelitis optica spectrum disorder and phenotype distribution. J Neurol 2009; 256: 1891–1898. 19479168 10.1007/s00415-009-5171-x

[24] Siritho S Nakashima I Takahashi T AQP4 antibody-positive Thai cases: Clinical features and diagnostic problems. Neurology 2011; 77: 827–834. 21813785 10.1212/WNL.0b013e31822c61b1 PMC3463102

[25] Kitley J Leite MI Nakashima I Prognostic factors and disease course in aquaporin-4 antibody-positive patients with neuromyelitis optica spectrum disorder from the United Kingdom and Japan. Brain 2012; 135: 1834–1849. 22577216 10.1093/brain/aws109

[26] Collongues N Marignier R Zephir H Long-term follow-up of neuromyelitis optica with a pediatric onset. Neurology 2010; 75: 1084–1088. 20855851 10.1212/WNL.0b013e3181f39a66

[27] Quek AM McKeon A Lennon VA Effects of age and sex on aquaporin-4 autoimmunity. Arch Neurol 2012; 69: 1039–1043. 22507888 10.1001/archneurol.2012.249 PMC3746965

[28] Collongues N Marignier R Jacob A Characterization of neuromyelitis optica and neuromyelitis optica spectrum disorder patients with a late onset. Mult Scler 2013; 20: 1086–1094. 24323817 10.1177/1352458513515085

[29] Kitley J Leite MI Küker W Longitudinally extensive transverse myelitis with and without aquaporin 4 antibodies. JAMA Neurol 2013; 70: 1375–1378. 23999580 10.1001/jamaneurol.2013.3890

[30] Marignier R Bernard-Valnet R Giraudon P Aquaporin-4 antibody-negative neuromyelitis optica: Distinct assay sensitivity-dependent entity. Neurology 2013; 80: 2194–2200. 23658379 10.1212/WNL.0b013e318296e917

[31] Downer JJ Leite MI Carter R Diagnosis of neuromyelitis optica (NMO) spectrum disorders: Is MRI obsolete? Neuroradiology 2012; 54: 279–285. 21553012 10.1007/s00234-011-0875-x

[32] Asgari N Skejoe HPB Lillevang ST Modifications of longitudinally extensive transverse myelitis and brainstem lesions in the course of neuromyelitis optica (NMO): A population-based, descriptive study. BMC Neurol 2013; 13: 33. 23566260 10.1186/1471-2377-13-33 PMC3622587

[33] Sepulveda M Blanco Y Rovira A Analysis of prognostic factors associated with longitudinally extensive transverse myelitis. Mult Scler 2013; 19: 742–748. 23037550 10.1177/1352458512461968

[34] Matsushita T Matsuoka T Isobe N Association of the HLA-DPB1*0501 allele with anti-aquaporin-4 antibody positivity in Japanese patients with idiopathic central nervous system demyelinating disorders. Tissue Antigens 2009; 73: 171–176. 19140826 10.1111/j.1399-0039.2008.01172.x

[35] Brum DG Barreira AA dos Santos AC HLA-DRB association in neuromyelitis optica is different from that observed in multiple sclerosis. Mult Scler 2010; 16: 21–29. 19995845 10.1177/1352458509350741

[36] Deschamps R Paturel L Jeannin S Different HLA class II (DRB1 and DQB1) alleles determine either susceptibility or resistance to NMO and multiple sclerosis among the French Afro- Caribbean population. Mult Scler 2011; 17: 24–31. 20861181 10.1177/1352458510382810

[37] Zephir H Fajardy I Outteryck O Is neuromyelitis optica associated with human leukocyte antigen? Mult Scler 2009; 15: 571–579. 19299434 10.1177/1352458508102085

[38] Matiello M Kim HJ Kim W Familial neuromyelitis optica. Neurology 2010; 75: 310–315. 20660861 10.1212/WNL.0b013e3181ea9f15 PMC2918892

